# METTL14-mediated m^6^A modification of circORC5 suppresses gastric cancer progression by regulating miR-30c-2-3p/AKT1S1 axis

**DOI:** 10.1186/s12943-022-01521-z

**Published:** 2022-02-14

**Authors:** Hui-Ning Fan, Zhao-Yu Chen, Xiao-Yu Chen, Ming Chen, You-Cai Yi, Jin-Shui Zhu, Jing Zhang

**Affiliations:** Department of Gastroenterology, Shanghai Jiao Tong University Affiliated Sixth People’s Hospital, Yishan Road 600, Shanghai, China

**Keywords:** m^6^A, METTL14, circORC5, miR-30c-2-3p, Gastric cancer

## Abstract

**Background:**

N6-methyladenosine (m^6^A) RNA methylation and circular RNAs (circRNAs) have been shown to act vital roles in multiple malignancies including gastric cancer (GC). However, there is little knowledge about how m^6^A modification of circRNAs contributes to GC progression.

**Methods:**

The association of METTL14 expression with the clinicopathological characteristics and prognosis in patients with GC was assessed by Western blot, Immunohistochemistry and public datasets. In vitro and vivo function experiments were conducted to investigate the role of METTL14 in GC. Furthermore, m^6^A-circRNA epitranscriptomic microarray was utilized to identify METTL14-mediated m^6^A modification of circRNAs, which were validated by methylated RNA immunoprecipitation (Me-RIP), RT-qPCR and rescue experiments in GC cells. The sponge of circORC5 with miR-30c-2-3p was confirmed by luciferase gene report and RNA immunoprecipitation assays. The expression, localization and prognosis of circORC5 in GC were evaluated by fluorescence in situ hybridization. The effects of METTL14 and (or) circORC5 on miR-30c-2-3p-mediated AKT1S1 and EIF4B were estimated by RT-qPCR and Western blot analyses.

**Results:**

We found that METTL14 was downregulated in GC tissue samples and its low expression acted as a prognostic factor of poor survival in patients with GC. Ectopic expression of METTL14 markedly repressed growth and invasion of GC cells *in vitro* and *in vivo*, whereas knockdown of METTL14 harbored the opposite effects. Mechanically, m^6^A-circRNA epitranscriptomic microarray and Me-RIP identified circORC5 as the downstream target of METTL14. Silencing of METTL14 reduced the m^6^A level of circORC5, but increased circORC5 expression. Moreover, circORC5 could sponge miR-30c-2-3p, and reverse METTL14-caused upregulation of miR-30c-2-3p and downregulation of AKT1S1 and EIF4B. In addition, circORC5 possessed a negative correlation with miR-30c-2-3p and indicated a poor survival in GC.

**Conclusion:**

Our findings demonstrate that METTL14-mediated m^6^A modification of circORC5 suppresses gastric cancer progression by regulating miR-30c-2-3p/AKT1S1 axis.

**Supplementary Information:**

The online version contains supplementary material available at 10.1186/s12943-022-01521-z.

## Introduction

Although the incidence and mortality of gastric cancer (GC) present a downward trend worldwide [1], but GC remains the third leading cause of cancer-related deaths in China [2]. Recent decades have witnessed the great progress in the treatment of GC including endoscopic resection, targeted therapy, and immunotherapy [3]. But, the prognosis of advanced patients is still poor owing to tumor invasion and metastasis. Thus, comprehending the molecular mechanisms of tumorigenesis is of importance to the early diagnosis and treatment of GC.

N6-methyladenosine (m^6^A) as one of the most common chemical modifications in eukaryotic mRNAs acts pivotal roles in cancer progression [4]. The m^6^A methylation can be catalyzed by the methyltransferases such as METTL3/14/16 (“writers”), removed by demethylases FTO and ALKBH5 (“erasers”), and interacts with m^6^A binding proteins, such as YTHDF1/2/3 and IGF2BP1/2/3 (“readers”) [5]. It has been shown that METTL14 acts as a tumor suppressor in colorectal cancer (CRC) [6, 7], bladder cancer [8] and breast cancer [9], but represents an oncogenic factor in thyroid cancer [10], pancreatic cancer [11] and leukemogenesis [12]. However, METTL14-mediated m^6^A modification in GC remains largely unknown.

Circular RNAs (circRNAs) as another subset of noncoding RNAs are characterized by closed ring structure and resistance to RNase R [13]. Increasing evidence shows that circRNAs can act as sponges of miRNAs to regulate cancer progression [14–16]. CircRNAs also function in GC by sponging miRNAs. For example, circRHOBTB3, circPSMC3, circCCDC9 and hsa_circ_0004872 inhibit growth and metastasis of GC by sponging miR-654-3p/-296-5p/-6792-3p/-224 [17–20], whereas circNRIP1 and circLMTK2 promotes GC progression by sponging miR-149-5p/-150-5p [21, 22]. Our previous studies demonstrated that circLARP4 and circYAP1 repress GC growth [23, 24], but circDLST boosts its progression [25]. However, how METTL14-mediated m^6^A modification of circRNAs contributes to GC is still elusive.

M^6^A-mediated modification of noncoding RNAs (ncRNAs) is implicated in cancer. METTL14 suppresses carcinogenesis by regulating m^6^A-dependent miR-375 processing or lncRNA X-inactive specific transcript (XIST) [6, 26]. m^6^A-modified circ-SORE promotes sorafenib resistance in hepatocellular carcinoma (HCC) by regulating β-catenin signaling [27]. Herein, we found that METTL14 suppressed growth and invasion of GC by regulating circORC5/miR-30c-2-3p axis and might provide a potential therapeutic target for GC.

## Methods

### Clinical data

Clinical and pathological data for 385 cases of GC patients and 33 paired tumor tissue samples were downloaded from The Cancer Genome Atlas database (http://xena.ucsc.edu/getting-started/). Clinicopathological characteristics including age, sex, stage, pathological stage, Tumor-Node-Metastasis (TNM) stage, survival and recurrence were also collected. 10 paired GC tissues were stored in liquid nitrogen and frozen at − 80 °C. A human tissue microarray containing 90 paired tumor tissues from GC patients (Lot No. XT14-008) was purchased from the Shanghai Outdo Biotech Company (Shanghai, China). Our study protocol was approved by the Ethics Committee of Shanghai Sixth People’s Hospital.

### Immunohistochemical analysis

The tissue microarray was deparaffinized, rehydrated, and microwaved-heated in sodium citrate buffer (10 mmol/L, pH6.0) for antigen retrieval. Then, the slides were incubated with Mouse anti-METTL14 monoclonal antibody (1:100, Lot No. CABT-B9471, NY, USA) by SABC (mouse IgG)—FITC immunohistochemical Kit (Lot. No. A0130, Wuhan, China). The protein expression of METTL14 (H score) was assessed by two independent pathologists. H-score = ΣPi(i + 1), where “Pi” represents the percentage of positive cells in all cells in the section, and “i” stands for coloring intensity.

### RNA fluorescence in situ hybridization (FISH)

The expression levels and cellular localization of hsa_circ_0007612 (circORC5) and hsa-miR-30c-2-3p in GC tissue samples were examined by FISH analysis. Digoxin-labeled probe sequence for circORC5 (Green fluorescence, 5’-ATTTTTCCATGATG CTTGCAATAGCTCT-3') and Biotin-labeled probe sequence for hsa-miR-30c-2-3p (Red fluorescence, 5’- AGAGTAAACAGCCTTCTCCCAG -3’) were synthesized for FISH analysis. The detailed description of FISH analysis was conducted as previously reported [23].

### RNA extraction and real-time quantitative PCR (RT-qPCR)

Total RNA was extracted using a RNA extraction kit (77,064, QIAGEN) and cDNA synthesis was performed using a reverse transcription kit (Promega, Madison, USA) according to the manufacturer’s instructions. PCR was conducted using the SYBR Green Master Mix (Q111-02, VAZYME). After the reactions were completed, relative gene expression level was calculated using the 2^−ΔΔCt^. The transfection efficiency of the vectors was defined by the extent of downregulation or upregulation of the target gene. The primer sequences used were indicated in Supplementary Table S1.

### Western blot

GC tissue samples and cells were lysed with RIPA buffer (P0013B, Beyotime Biotech, Shanghai, China). The supernatants were resolved in SDS-PAGE and transferred onto polyvinylidene fluoride (PVDF) membranes (IPVH00010, Millipore, MA, USA). The membranes were probed with anti-METTL14 (1:1000, CABT-B9471, NY, USA), anti-AKT1S1 (1:1000, AF7929, Affinity), anti-EIF4B (1:2000, 17917-1-AP, Wuhan, China) and anti-GAPDH (1:1000, AB-P-R 001, Hangzhou, China) overnight at 4°C. Protein bands were shown by enhanced chemiluminescence (ECL) method.

### Plasmid, siRNA, miRNA mimic and inhibitor

METTL14 Plasmid vector, siRNA targeting METTL14 (si-METTL14, 5’-CCGACAGCATTGGTGCCGTGTTAAA-3’) or circORC5 (si-circORC5, 5’- AGCTATTGCAAGCATCATGGA-3’), miR-30c-2-3p mimics and inhibitor were purchased from GenePharma (Shanghai, China). The negative vector, si-NC, and miR-NC were indicated as the control groups. GC cell lines were planted in 6-well plates 24 h prior to si-METTL14, si-circORC5, miR-30c-2-3p mimic or inhibitor transfection with 50–60% confluence, and then mixed with Lipofectamine 2000 (Invitrogen, Carlsbad, CA, USA) according to the manufacture instructions.

### Cell culture, MTT, colony formation, Transwell assays, RNase R treatment and Nuclear and cytoplasmic fractionation

These assays were conducted as previously reported [23–25].

### Human m^6^A-circRNA epitranscriptomic microarray

Total RNA from each sample was quantified using the NanoDrop ND-1000. The sample preparation and microarray hybridization were performed based on the Arraystar’s standard protocols. Briefly, the total RNAs were immunoprecipitated with anti- m^6^A antibody (Nano materials intestinal injury). The modified RNAs were eluted from the immunoprecipitated magnetic beads as the “IP”. The unmodified RNAs were recovered from the supernatant as “Sup”. The “IP” and “Sup” RNAs were treated with RNase R, and then labeled with Cy5 and Cy3 respectively as cRNAs in separate reactions using Arraystar Super RNA Labeling Kit. The cRNAs were combined together and hybridized onto Arraystar Human circRNA Epi-transcriptomic Microarray. After washing the slides, the arrays were scanned in two-color channels by an Agilent Scanner G2505C.

### RNA immunoprecipitation (RIP) and m^6^A RIP (MeRIP)

The RIP assay was executed using RNA-Binding Protein Immunoprecipitation Kit (17–700, Millipore) and HiScript II qRT SuperMix II kit (R223, VAZYME). Anti-m6A antibody (A-1801–020, Epigentek) and anti-Ago2 (ab5072, Cambridge, MA, USA) were used for RIP and MeRIP assays.

### Bioinformatic analysis

The circORC5 specific binding with miRNAs (miR-137, miR-374a-5p, miR-140-5p, miR-30c-2-3p and miR-9-5p) was identified using the m^6^A-circRNA profiling and miRbase database (http://www.mirbase.org /index.shtml). The targets of miR-30c-2-3p were identified using TargetScanHuman7.1 (http://www.targetscan.org/vert_71/).

### Luciferase reporter assay

Luciferase reporter assay was conducted using Double luciferase reporter gene detection kit (RG027, Beyotime). MGC-803 and AGS cells were seeded into 96-well plates and co-transfected with PRL-TK-pMIR-circORC5 or PRL-TK-pMIR-AKTS1/EIF4B 3’UTR, and miR-30c-2-3p mimics or miR-NC. After 48 h of incubation, the firefly and Renilla luciferase activities were examined with a dual-luciferase reporter assay.

### In vivo tumor growth assay

Male nude mice (6 weeks old) were purchased from the Shanghai Laboratory Animal Central (SLAC, Shanghai, China). SGC-7901 cells (1 × 10^7^) transfected with the METTL14 overexpression vector or no-load lentivirus vector were resuspended in 200μL of sterile PBS and injected subcutaneously into the right flanks of mice. After 4 weeks, the mice were sacrificed, and the xenografted tumors were collected for hematoxylin–eosin (HE) staining and IHC analysis. Tumor volume was calculated using formula: volume = (length x width^2^)/2. The animal experiments were approved by the Ethics Committee of Shanghai Sixth People’s Hospital.

### Statistical analysis

Statistical analysis was performed with GraphPad Prism 7 (La Jolla, CA, USA). In brief, the values are expressed as the mean ± standard deviation (SD). Student’s test and analysis of variance were used for comparisons between groups. Kaplan–Meier analysis was used to assess the association of circORC5 or miR-30c-2-3p with GC prognosis. A Cox proportional hazard model was used to assess the risk of circORC5 or miR-30c-2-3p in GC. Pearson Correlation Analysis was used to analyze the correlation of circORC5 with miR-30c-2-3p. The categorical data were analyzed by chi-square of Fisher’s exact tests. P < 0.05 was considered statistically significant.

## Results

### Downregulation of METTL14 predicted poor prognosis in patients with GC

**Fig. 1:**
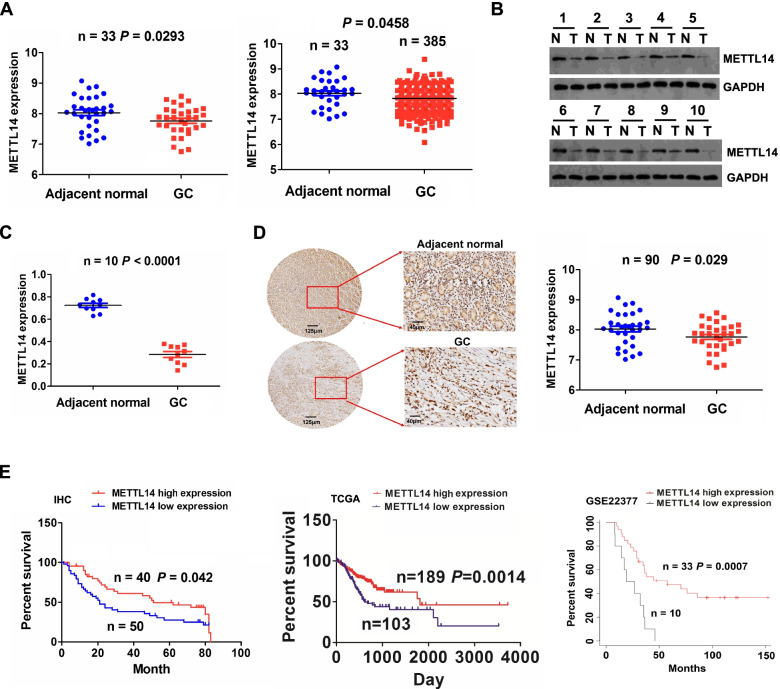
Downregulation of METTL14 was associated with poor prognosis in GC. **A** TCGA assessment of the expression levels of METTL14 in paired and unpaired GC tissues. **B, C** Western blot analysis of the protein expression of METTL14 in 10 paired GC tissue samples. **D** IHC analysis of the protein expression of METTL14 in 90 paired GC tissue samples. **E** Tissue microarray as well as TCGA and GSE22377 cohorts analysis of the association of METTL14 with the prognosis of GC patients

To investigate the potential role of METTL14 in GC, we first analyzed the RNA sequencing data from TCGA, which showed decreased METTL14 expression in human GC tissue samples relative to normal tissue (Fig. 1A, *P* < 0.05). We further detected the protein levels of METTL14 in 10 paired GC tissue samples by Western blot, which indicated that METTL14 was remarkably downregulated in GC compared to paired normal samples (Fig. 1B, C, *P* < 0.0001). Tissue microarray containing 90 pairs of cancerous and matched normal tissue was analyzed by IHC staining, which further validated the downregulation of METTL14 in GC tissues (Fig. 1D, *P* = 0.029).

Then, we found that downregulation of METTL14 showed no association with clinicopathological characteristics including pathological stage and TNM stage in patients with GC (Supplementary Table S2), however, Kaplan–Meier analysis uncovered that the patients with low-METTL14 expression indicated poor overall survival as compared with those with high-METTL14 expression (Fig. 1E, *P* = 0.042). Moreover, independent two cohorts were established by extracting TCGA and clinical data of GC patients from GSE22377. In coinciding with our results, low expression of METTL14 harbored worse survival in GC (Fig. 1E, *P* < 0.01). In addition, multi-variate analysis revealed that METTL14 low expression as well as age and pathological stage was a risk factor for poor survival in patients with GC from TCGA cohort (Supplementary Table S3, *P* = 0.005). These results suggested that METTL14 was a reliable prognostic factor in patients with GC.

### Knockdown of METTL14 facilitated proliferation and invasion of GC

**Fig. 2:**
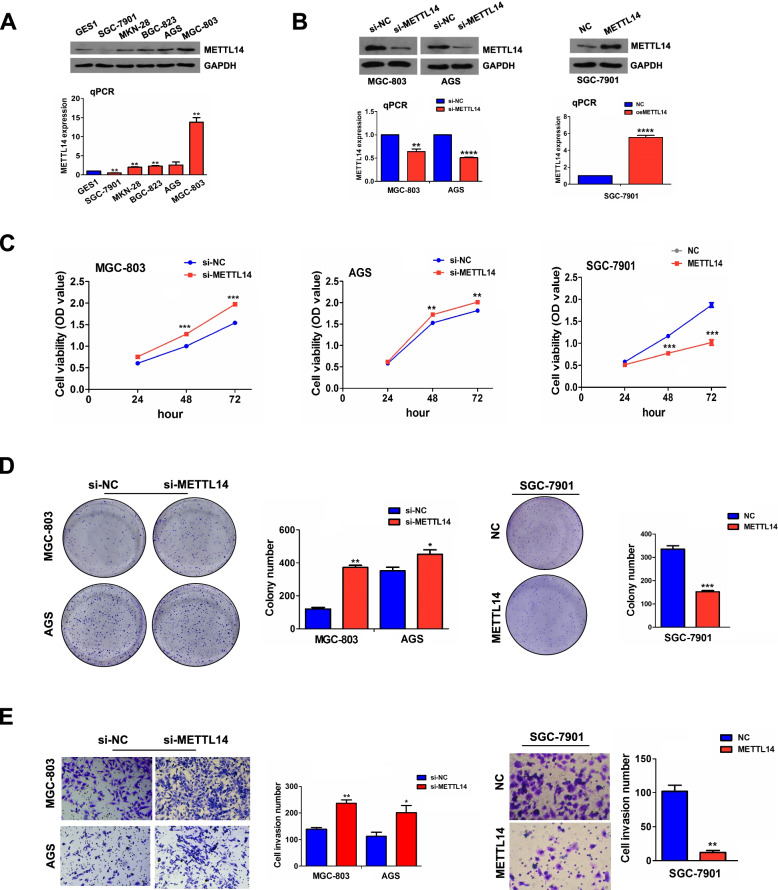
Knockdown of METTL14 promoted proliferation and invasion of GC. **A** RT-qPCR and Western blot analysis of the mRNA and protein expression levels of METTL14 in GSE1 and GC cell lines. **B** RT-qPCR and Western blot analysis of the transfection efficiency of si-METTL14 in MGC-803 and AGS cells or METTL14 in SGC-7901 cells. **C** MTT analysis of the effects of METTL14 knockdown or overexpression on the GC cell viability. **D** Colony formation analysis of the effects of METTL14 knockdown or overexpression on the colony formation ability. **E** Transwell analysis of the effects of METTL14 knockdown or overexpression on the GC cell invasion. Data are the means ± SEM of three experiments. *P < 0.05; **P < 0.01; ***P < 0.001; ****P < 0.0001.

Given the fact that METTL14 expression is decreased in GC tissues, we speculated that METTL14 may function as a tumor suppressor in GC. We detected the mRNA and protein levels of METTL14 in normal gastric epithelial cell line (GES1) and GC cell lines (SGC-7901, MKN-28, BGC-823, AGS and MGC-803) by RT-qPCR and Western blot, and found that METTL14 possessed high expression in AGS and MGC-803, but low expression in SGC-7901 (Fig. 2A). Therefore, to confirm the roles of METTL14 in GC, we established METTL14-kockdown cell model in AGC and MGC-803 cells with siRNA, and METTL14 overexpression cell model in SGC-7901 cells with plasmids. The transfection efficiency was determined by RT-qPCR and Western blot (Fig. 2B). The cell viability and proliferative capabilities of GC cells were then assessed by MTT and colony formation assay. As expected, knockdown of METTL14 remarkably promoted cell viability (Fig. 2C) and proliferative potential (Fig. 2D). In addition, Transwell invasion assay indicated that downregulation of METTL14 dramatically facilitated invasion capabilities of GC cells (Fig. 2E). Likewise, overexpressed METTL14 exhibited inhibitory effects on cell viability (Fig. 2C), colony formation (Fig. 2D) and invasion capabilities (Fig. 2E) relative to the control group.

### METTL14 acted by m6A-dependent modification of circORC5

**Fig. 3:**
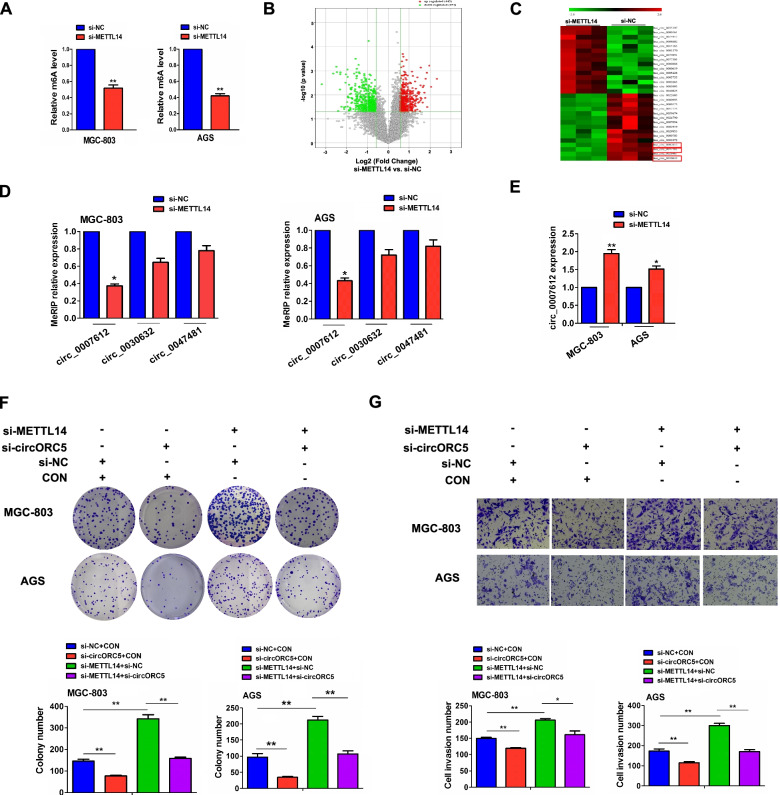
METTL14 acted by m^6^A-dependent modification of circORC5. **A** MeRIP analysis of the effects of METTL14 knockdown on total m^6^A levels in MGC-803 and AGS cells. **B, C** Volcano plots and cluster mapping of the differential circRNAs in m^6^A levels by m^6^A-circRNA epitranscriptomic microarray. **D** MeRIP analysis of the m^6^A levels of circORC5, circ_0030632 an circ_0047481 in METTL14-knockdown MGC-803 and AGS cells. **E** RT-qPCR analysis of circORC5 expression in METTL14-knockdown MGC-803 and AGS cells. **F** Colony formation and **G** Transwell invasion assay were used to assess the effects of transfection with si-circORC5 and (or) si-METTL14 on cell proliferation and invasion in MGC-803 and AGS cells. Data are the means ± SEM of three experiments. *P < 0.05; **P < 0.01

To elucidate the underlying mechanisms of METTL14 in GC progression, we first detected the effects of METTL14 on the m^6^A level in MGC-803 and AGS cells by MeRIP, which showed that the m^6^A level was markedly decreased in METTL14-knockdown MGC-803 and AGS cells (Fig. 3A). Then, m^6^A-circRNA epitranscriptomic microarray revealed that 444 circRNAs increased in m^6^A levels but 454 decreased in m^6^A levels in METTL14-knockdown MGC-803 cells compared with the control group (Fig. 3B), among which, hsa_circ_0030632, hsa_circ_0047481 and hsa_circ_007612 were the top three downregulated circRNAs in m^6^A levels in METTL14-knockdown cells (Fig. 3C). MeRIP-PCR further validated that the m^6^A level of hsa_circ_0007612 (circORC5) rather than hsa_circ_0030632 and hsa_circ_0047481 was decreased by knockdown of METTL14 in MGC-803 and AGS cells (Fig. 3D), whereas RT-qPCR analysis showed that circORC5 mRNA level was increased by METTL14 knockdown in MGC-803 and AGS cells (Fig. 3E).

The transfection efficiency of si-circORC5 in MGC-803 and AGS cells was determined by RT-qPCR analysis (Supplementary Fig. S1). Functionally, we found that circORC5 depletion reduced colony formation and invasion capabilities, and counteracted si-METTL14 induced colony formation and invasion potential in MGC-803 and AGS cells (Fig. 3F, G). These results suggested that METTL14 acted by m^6^A-dependent modification of circORC5.

### Identification and characteristics of circORC5 in GC

**Fig. 4:**
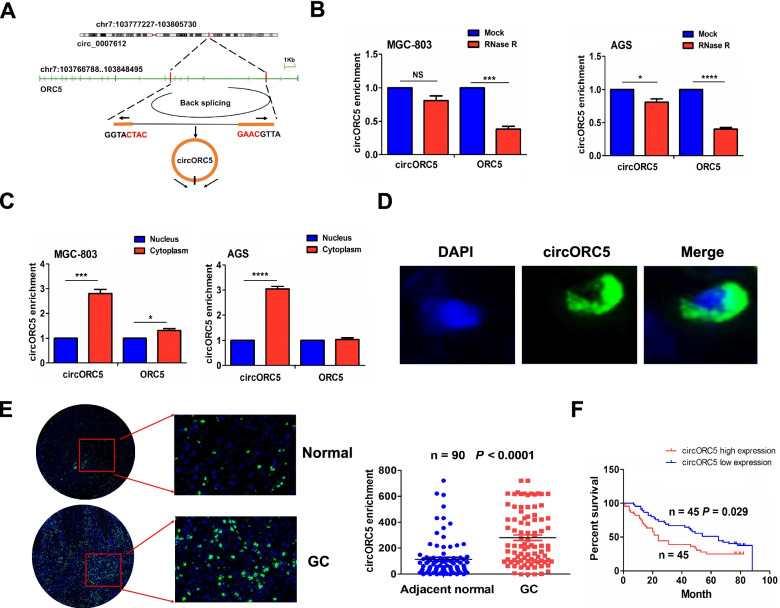
Identification and characteristics of circORC5. **A** The genomic loci of the ORC5 gene and circORC5. **B** RT-qPCR analysis of circORC5 stability after exposure to treatment with RNase R exonuclease in MGC-803 and AGS cells. **C** RT-qPCR analysis of the cytoplasmic or nuclear location of circORC5 in MGC-803 and AGS cells. **D** FISH assessment of circORC5 localization in GC cells. **E** FISH analysis of the expression of circORC5 in 90 paired GC tissue samples. **F** Kaplan–Meier plotter of the association of circORC5 with overall survival in patient with GC. Data are the means ± SEM of three experiments. *P < 0.05; ***P < 0.001; ****P < 0.0001

According to the circRNA annotation in Circular RNA interactome (https:// circinteractome.nia.nih.gov/index.html), hsa_circ_0007612 is derived from the linear gene origin recognition complex subunit 5 (ORC5) and termed as circORC5 (Fig. 4A). Compared with linear ORC5, circORC5 harbored higher stability after treatment with RNase R exonuclease (Fig. 4B). Cytoplasmic and nuclear RNA analysis showed that circORC5 was preferentially localized in the cytoplasm in MGC-803 and AGS cells (Fig. 4C). FISH further validated that green fluorescent distribution of circORC5 was mainly in the cytoplasm of GC (Fig. 4D), and circORC5 had increased expression in GC tissues compared with the adjacent normal (Fig. 4E). Kaplan–Meier unveiled that GC patients with circORC5 high expression harbored poorer survival as compared with those with circORC5 low expression (Fig. 4F).

### CircORC5 had a negative correlation with miR-30c-3p in GC

**Fig. 5:**
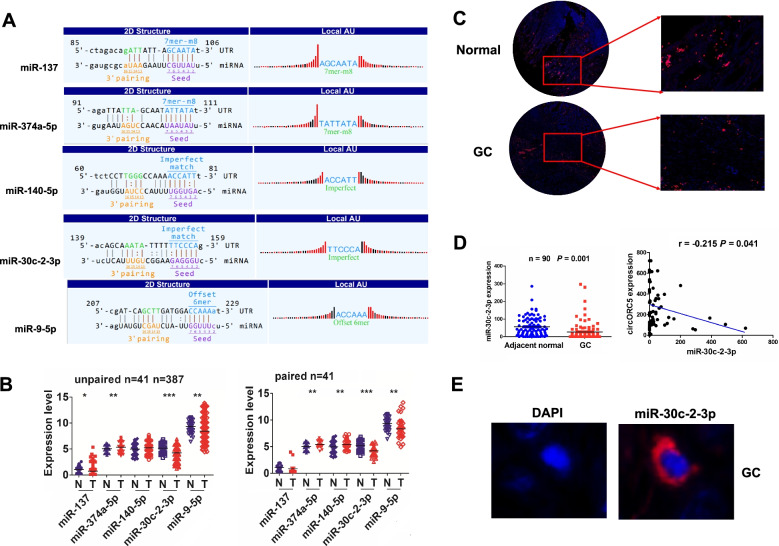
CirORC5 harbored a negative correlation with miR-30c-2-3p in GC. **A** The potential target miRNAs sponged by cirORC5 were identified by m6A-circRNA profiling and miRbase. **B** The expression levels of these five miRNAs in both 387 unpaired and 41 paired GC tissues were analyzed by TCGA. **C, D** FISH analysis was performed to validate the expression levels of miR-30c-2-3p and its correlation with circORC5 expression in 90 paired GC tissues. **E** FISH analysis of the cellular localization of miR-30c-2-3p in GC tissues

According to m^6^A-circRNA profiling and miRbase, cirORC5 was identified to have the potential to bind with five miRNAs (Fig. 5A). We analyzed the expression levels of these five miRNAs in GC and found that miR-30c-2-3p possessed most significant decrease in 387 unpaired and 41 paired GC tissues (Fig. 5B, *P* < 0.0001). FISH analysis further validated that miR-30c-2-3p expression levels were markedly downregulated and negatively correlated with circORC5 in GC tissues (Fig. 5C, D). FISH analysis indicated that circORC5 was also localized in the cytoplasm of GC tissue (Fig. 5E).

### CircORC5 acted as a sponge of miR-30c-2-3p in GC

**Fig. 6:**
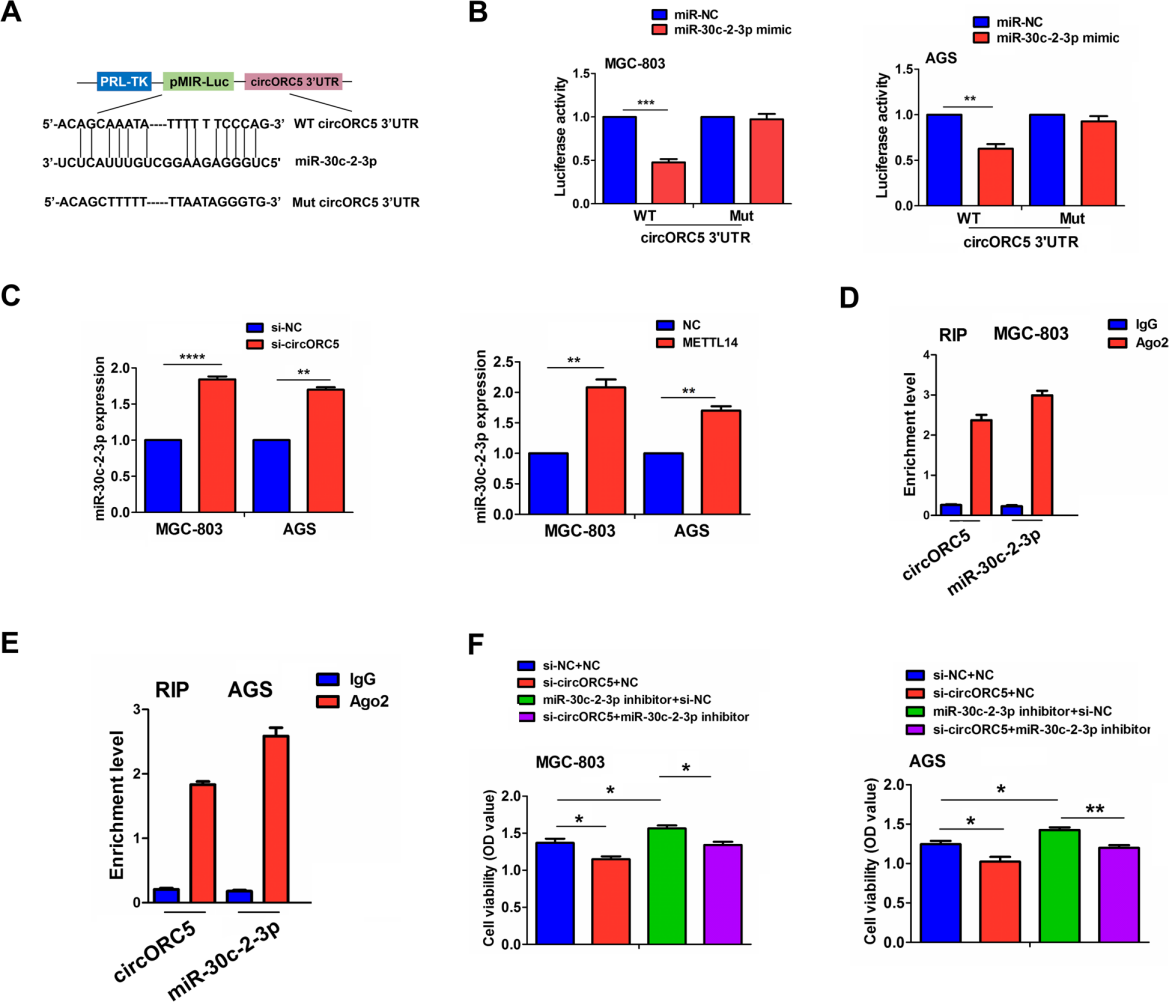
CircORC5 acted as a sponge of miR-30c-2-3p in GC. **A** Schematic representation of potential binding sites between circORC5 and miR-30c-2-3p. **B** The luciferase activity of the WT luc-circORC5 or Mut luc-circORC5 after transfection with miR-30c-2-3p mimics in MGC-803 and AGS cells. **C** RT-qPCR was performed to observe the effects of circORC5 knockdown or METTL14 overexpression on miR-30c-2-3p expression in the MGC-803 and AGS cells. **D, E** RIP for Ago2 was performed to examine the endogenous expression of circORC5 and miR-30c-2-3p in MGC-803 and AGS cells. **F** MTT analysis of the effect of transfection with si-circORC5 and (or) miR-30–2-3p inhibitor on the cell viability in MGC-803 and AGS cells. Data are the means ± SEM of three experiments. *P < 0.05; **P < 0.01; ***P < 0.001; ****P < 0.0001

The binding sites of miR-30c-2-3p with circORC5 are demonstrated in Fig. 6A. We found that miR-30c-2-3p mimics could reduce the luciferase activity of the WT circORC5 3’-untranslated region (UTR), but had no effect on that of the Mut circORC5 3’-UTR compared with the miR-NC group in MGC-803 and AGS cells (Fig. 6B). RT-qPCR analysis indicated that the expression of miR-30c-2-3p was remarkably increased by circORC5 knockdown or METTL14 overexpression in MGC-803 and AGS cells (Fig. 6C), but miR-30c-2-3p mimics showed no effects on circORC5 expression (Supplementary Fig. S2). Furthermore, we performed RNA immunoprecipitation (RIP) for Ago2 in MGC-803 and AGS cells and investigated the endogenous levels of circORC5 and miR-30c-2-3p pulled-down from Ago2-expressed cells by RT-qPCR analysis, which indicated that circORC5 and miR-30c-2-3p were highly enriched in the Ago2 pellet compared with those in the input control (Fig. 6D, E). Functionally, miR-30c-2-3p inhibitor promoted cell viability and reversed circORC5 knockdown-induced anti-proliferative effect in MGC-803 and AGS cells (Fig. 6F).

### METTL14 mediated circORC5 to regulate miR-30c-2-3p/AKT1S1 axis

**Fig. 7:**
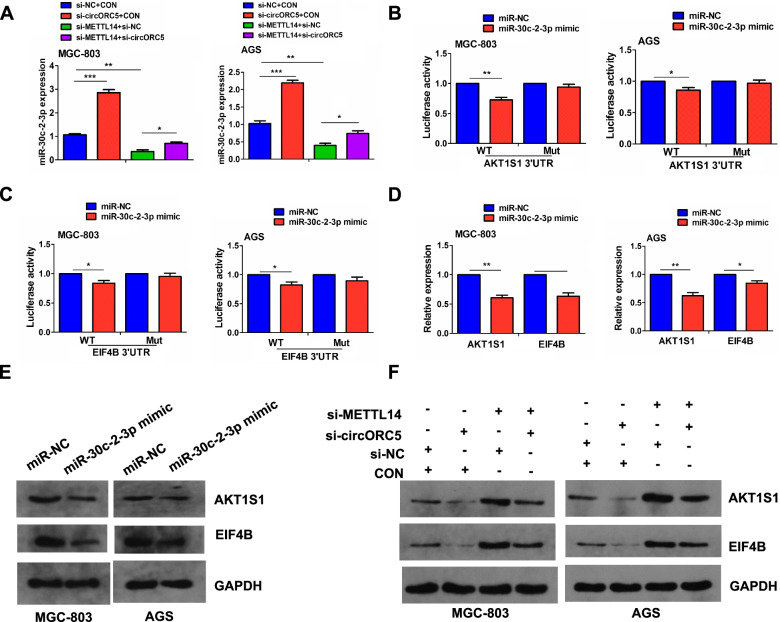
METTL14 mediated circORC5 to regulate miR-30c-2-3p/AKT1S1 axis. **A** RT-qPCR analysis of the effects of transfection with si-METTL14 and (or) si-circORC5 on miR-30c-2-3p expression in MGC-803 and AGS cells. **B** The luciferase activity of the WT luc-AKT1S1 or luc-AKT1S1 after transfection with miR-30c-2-3p mimics in MGC-803 and AGS cells. **C** The luciferase activity of the WT luc-EIF4B or luc-EIF4B after co-transfection with miR-30c-2-3p mimics in MGC-803 and AGS cells. **D** RT-qPCR **E** Western blot analysis of the effects of transfection with miR-30c-2-3p mimic on AKT1S1 and EIF4B expression in MGC-803 and AGS cells. **F** Western blot analysis of the effects of transfection with si-METTL14 and (or) si-circORC5 on AKT1S1 and EIF4B expression in MGC-803 and AGS cells. Data are the means ± SEM of three experiments. *P < 0.05; **P < 0.01; ***P < 0.001

To verify whether METTL14 mediated circORC5 to regulate miR-30c-2-3p expression, RT-qPCR analysis indicated that knockdown of circORC5 increased miR-30c-2-3p expression and reversed the inhibitory effect of METTL14 knockdown on miR-30c-2-3p expression in MGC-803 and AGS cells (Fig. 7A). Next, AKT1 substrate 1 (AKT1S1) and eukaryotic translation initiation factor 4B (EIF4B) were identified as the targets of miR-30c-2-3p, and schematic representation of potential binding sites of miR-30c-2-3p with AKT1S1/EIF4B was listed in Supplementary Fig. S3. We further found that miR-30c-2-3p mimics could reduce the luciferase activity of the WT AKT1S1 and EIF4B 3’ UTR, but had no effect on that of the Mut AKT1S1 and EIF4B 3’UTR compared with the miR-NC group in MGC-803 and AGS cells (Fig. 7B, C). Meanwhile, miR-30c-2-3p mimics could downregulate the expression of AKT1S1 and EIF4B (Fig. 7D, E). Further Western blot indicated that METTL14 knockdown considerably upregulated AKT1S1 and EIF4B expression in MGC-803 and AGC cells, and this effect could be reversed by circORC5 knockdown (Fig. 7E).

### METTl14 inhibited in vivo tumor growth

**Fig. 8:**
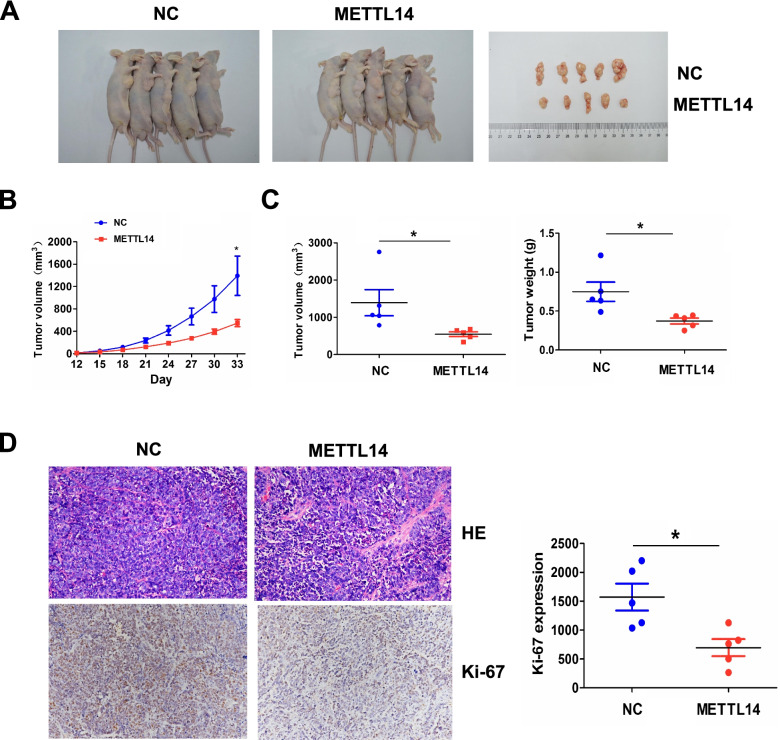
METTl14 inhibited in vivo tumor growth. **A** Comparison of the tumor size between METTL14 overexpression and NC transfected groups. **B** A growth curve analysis of the tumor growth in METTL14 overexpression and NC transfected groups. **C** Comparison of the tumor volume and weight between METTL14 overexpression and NC transfected groups. **D** H&E and IHC analysis of the cell proliferation and Ki-67 expression in METTL14 overexpression and NC transfected groups. Data are the means ± SEM of five experiments. *P < 0.05

To elucidate whether METTl14 suppressed in vivo tumor growth, we established a METTl14 or NC stably transfected SGC-7901 cell line, which was then subcutaneously injected into the flank of nude mice. After an observation for 33 days, we found that, the volumes of xenograft tumors induced by METTl14 transfected SGC-7901 cells were smaller than those by NC transfected cells (Fig. 8A). The growth curve demonstrated that, the tumors in METTl14 transfected group presented a reduction in a time dependent manner (Fig. 8B), and the tumor volume and weight were alleviated in METTl14 transfected group compared with the NC group (Fig. 8C). HE and IHC staining indicated that the tumor proliferation marker Ki-67 was downregulated in METTl14 transfected group compared with the NC group (Fig. 8D).

## Discussion

Accumulating data indicate that METTL14 as a m^6^A “writer” shows a decreased expression in CRC [7, 26], bladder cancer [8] and breast cancer [9], but an increased level in thyroid and pancreatic cancers [10, 11]. Downregulation of METTL14 predicts a poor prognosis in patients with CRC and breast cancer [7, 9, 26]. METTL14 has been reported to be downregulated in GC [28], but its clinical implication in patients with GC is unknown. We herein found that, in coinciding with previous studies, METTL14 levels were lowered in GC and low expression of METTL14 was a prognostic factor of poor survival in patients with GC. According to the previous studies, the loss of METTL14 in tumor-associated macrophages promotes tumor growth [29] and SUMOylation of the m^6^A-RNA methyltransferase influences its function [30]. These reports may explain why METTL14 is downregulated in cancer but need be further studied in GC.

Functionally, METTL14 has dual roles in cancer. It can repress growth, invasion and metastasis in multiple malignancies [6–9, 26], but accelerate tumor progression [10, 11]. A previous study showed that METTL14 could suppress the proliferation and invasion by inhibiting the PI3K/AKT/mTOR signaling [28]. Herein, we also found that knockdown of METTL14 promoted the growth and invasion of GC cells in vitro and in vivo, whereas overexpressing METTL14 harbored the opposite effects. These studies suggested that METTL14 might be a tumor suppressor in GC.

Mechanically, METTL14 participates in tumorigenesis not only by m^6^A-dependent modification of mRNAs [7, 8, 11], but also by modification of miR-375 or lncRNA XIST [6, 26]. Previous report indicated that circGFRα1 mediated by METTL14 facilitates self-renewal of female germline stem cells [31]. Herein, m^6^A-circRNA profiling and Me-RIP were utilized to identify that METTL14 could exhibit m^6^A-dependent modification of circORC5 in GC cells. Downregulation of METTL14 decreased the m^6^A level of circORC5, but increased circORC5 expression. Knockdown of circORC5 repressed the colony formation and invasive capabilities, and attenuated METTL14 loss-induced tumor promoting effects in GC cells. Our findings unveiled that METTL14 might act by m^6^A-dependent modification of circORC5.

It is known that circRNAs act as the sponges of miRNAs implicated in GC progression [17–22]. Our previous studies indicated that circLARP4 or circDLST can act as a sponge of miR-424-5p or miR-502-5p to impact growth and invasion of GC [23, 25]. Herein, we found that circORC5 was upregulated in GC tissue samples and associated with poor survival in patents with GC. CircORC5 could bind with miR-30c-2-3p and decrease its expression in GC cells. It has been reported that miR-30c-2-3p is downregulated in pancreatic ductal adenocarcinoma [32] and breast cancer [33], and represses cell proliferation and cycle progression [33, 34]. We also found that miR-30c-2-3p was downregulated in GC tissue samples, possessed a negative correlation with circORC5 expression and reversed circORC5-induced cell proliferation in GC cells. METTL14 deficiency decreased miR-30c-2-3p expression while downregulation of circORC5 increased its expression. LINC00346 and hsa_circ_0072995 can also sponge miR-30c-2-3p to promote tumor growth [35, 36]. Our findings uncovered that circORC5 mediated by METTL14 promoted GC growth by sponging miR-30c-2-3p.

**Fig. 9:**
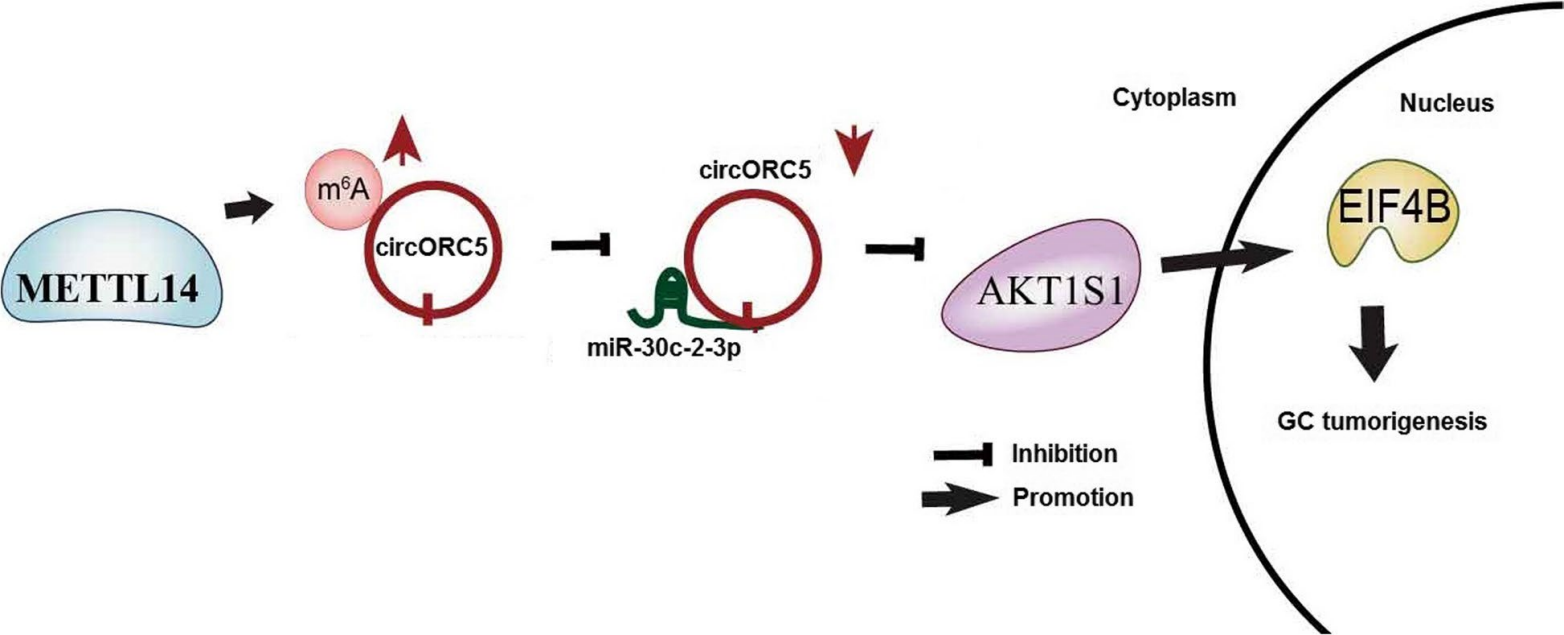
Schematic representation of proposed mechanism of METTL14 in GC. METTL14 increased the m^6^A levels of circORC5 and inhibited circORC5 expression, thereby upregulated miR-30c-2-3p and downregulated AKT1S1 and EIF4AB, contributing to suppression of GC progression

It has been shown that RAB31, member RAS oncogene family as a target of miR-30c-2-3p favors GC cell proliferation [37]. We also identified AKT1S1 and EIF4B as the direct targets of miR-30c-2-3p in GC cells. AKT1S1 is upregulated in HCC, associates with poor prognosis in patients with HCC and promotes HCC growth [38]. ElF4B-PI3K-AKT signaling promotes proliferation and inhibits apoptosis in nasopharyngeal carcinoma [39]. Herein, we found that miR-30c-2-3p or knockdown of circORC5 decreased the expression of ElF4B and AKT1S1 and circORC5 deficiency reversed the promoting effects of METTL14 knockdown on ElF4B and AKT1S1 expression in GC cells. Our findings indicated that, METTL14 suppressed GC growth by increasing the m^6^A level of circORC5 and decreasing its expression, and circORC5 sponged miR-30c-2-3p and upregulated ElF4B and AKT1S1, contributing to GC tumorigenesis (Fig. 9).

In conclusion, our findings demonstrate that downregulation of METTL14 was associated poor survival in patients with GC, and METTL14-mediated m^6^A modification of circORC5 inhibits growth and invasion of GC cells by regulating miR-30c-2-3p/AKT1S1 axis and may provide a promising prognostic factor for GC.

## Supplementary Information

**Additional file 1:** **Table S1.** The sequences of the primers. **Table S2.** The correlation of METTL14 expression with clinicopathologic characteristics of GC patients. **Table S3. **Cox regression analysis of METTL14 expression as survival predictor.

** Additional file 2: Supplementary Figure S1. **RT-qPCR analysis of the transfection efficiency of si-circORC5 in MGC-803 and AGS cells.

** Additional file 3: Supplementary Figure S2.** The transfection efficiency of miR-30c-2-3p mimics and its effects on circORC5 expression were measured by RT-qPCR in MGC-803 and AGS cells.

** Additional file 4: Supplementary Figure S3.** Schematic representation of potential binding sites between miR-30c-2-3p and AKT1S1/ EIF4B.

## Data Availability

All data generated or analysed during this study are included in this published article and its additional files.

## Abbreviations

m6A: N6-methyladenosine
METTL3/14/16: Methyltransferase 3/1416
ALKBH5: AlkB homolog 5, RNA demethylase
AKT1S1: AKT1 substrate
EIF4B: Eukaryotic translation initiation factor 4B
YTHDF1/2/3: YTH N6-methyladenosine RNA binding protein 1/2/3
RIP: RNA immunoprecipitation
TCGA: The Cancer Genome Atlas
FISH: Fluorescence in situ hybridization
MiRNA: MicroRNA
HCC: Hepatocellular carcinoma
GC: Gastric cancer
CircRNA: Circular RNA
TMA: Tissue microarray
FBS: Fetal bovine serum
IOD: Immunofluorescence accumulation optical density
qRT-PCR: Quantitative real-time PCR
TNM: Tumor-Node-Metastasis

## References

[1] Siegel RL, Miller KD, Jemal A (2020). Cancer statistics, 2020. CA Cancer J Clin.

[2] Chen W, Zheng R, Zhang S, Zeng H, Xia C, Zuo T, Yang Z, Zou X, He J (2017). Cancer incidence and mortality in China, 2013. Cancer Lett.

[3] Hashimoto T, Kurokawa Y, Mori M, Doki Y (2018). Update on the treatment of gastric cancer. JMA J.

[4] Chen XY, Zhang J, Zhu JS (2019). The role of m6A RNA methylation in human cancer. Mol Cancer.

[5] Lan Q, Liu PY, Haase J, Bell JL, Hüttelmaier S, Liu T (2019). The Critical Role of RNA m6A Methylation in Cancer. Cancer Res.

[6] Chen X, Xu M, Xu X, Zeng K, Liu X, Sun L, Pan B, He B, Pan Y, Sun H (2020). METTL14 Suppresses CRC Progression via Regulating N6-Methyladenosine-Dependent Primary miR-375 Processing. Mol Ther.

[7] Chen X, Xu M, Xu X, Zeng K, Liu X, Pan B, Li C, Sun L, Qin J, Xu T, He B, Pan Y, Sun H, Wang S (2020). METTL14-mediated N6-methyladenosine modification of SOX4 mRNA inhibits tumor metastasis in colorectal cancer. Mol Cancer.

[8] Gu C, Wang Z, Zhou N, Li G, Kou Y, Luo Y, Wang Y, Yang J, Tian F (2019). Mettl14 inhibits bladder TIC self-renewal and bladder tumorigenesis through N6-methyladenosine of Notch1. Mol Cancer.

[9] Gong PJ, Shao YC, Yang Y, Song WJ, He X, Zeng YF, Huang SR, Wei L, Zhang JW (2020). Analysis of N6-Methyladenosine Methyltransferase Reveals METTL14 and ZC3H13 as Tumor Suppressor Genes in Breast Cancer. Front Oncol.

[10] Zhang X, Li D, Jia C, Cai H, Lv Z, Wu B (2021). METTL14 promotes tumorigenesis by regulating lncRNA OIP5-AS1/miR-98/ADAMTS8 signaling in papillary thyroid cancer. Cell Death Dis.

[11] Wang M, Liu J, Zhao Y, He R, Xu X, Guo X, Li X, Xu S, Miao J, Guo J (2020). Upregulation of METTL14 mediates the elevation of PERP mRNA N6 adenosine methylation promoting the growth and metastasis of pancreatic cancer. Mol Cancer.

[12] Weng H, Huang H, Wu H, Qin X, Zhao BS, Dong L, Shi H, Skibbe J, Shen C, Hu C (2018). METTL14 inhibits hematopoietic stem/progenitor differentiation and promotes leukemogenesis via mRNA m6A modification. Cell Stem Cell.

[13] Memczak S, Jens M, Elefsinioti A, Torti F, Krueger J, Rybak A, Maier L, Mackowiak SD, Gregersen LH, Munschauer M (2013). Circular RNAs are a large class of animal RNAs with regulatory potency. Nature.

[14] Li J, Huang C, Zou Y, Ye J, Yu J, Gui Y (2020). CircTLK1 promotes the proliferation and metastasis of renal cell carcinoma by sponging miR-136-5p. Mol Cancer.

[15] Lu Q, Liu T, Feng H, Yang R, Zhao X, Chen W, Jiang B, Qin H, Guo X, Liu M (2019). Circular RNA circSLC8A1 acts as a sponge of miR-130b/miR-494 in suppressing bladder cancer progression via regulating PTEN. Mol Cancer.

[16] Zeng K, Chen X, Xu M, Liu X, Hu X, Xu T, Sun H, Pan Y, He B, Wang S (2018). CircHIPK3 promotes colorectal cancer growth and metastasis by sponging miR-7. Cell Death Dis.

[17] Deng G, Mou T, He J, Chen D, Lv D, Liu H, Yu J, Wang S, Li G (2020). Circular RNA circRHOBTB3 acts as a sponge for miR-654-3p inhibiting gastric cancer growth. J Exp Clin Cancer Res.

[18] Rong D, Lu C, Zhang B, Fu K, Zhao S, Tang W, Cao H (2019). CircPSMC3 suppresses the proliferation and metastasis of gastric cancer by acting as a competitive endogenous RNA through sponging miR-296-5p. Mol Cancer.

[19] Luo Z, Rong Z, Zhang J, Zhu Z, Yu Z, Li T, Fu Z, Qiu Z, Huang C (2020). Circular RNA circCCDC9 acts as a miR-6792-3p sponge to suppress the progression of gastric cancer through regulating CAV1 expression. Mol Cancer.

[20] Ma C, Wang X, Yang F, Zang Y, Liu J, Wang X, Xu X, Li W, Jia J, Liu Z (2020). Circular RNA hsa_circ_0004872 inhibits gastric cancer progression via the miR-224/Smad4/ADAR1 successive regulatory circuit. Mol Cancer.

[21] Zhang X, Wang S, Wang H, Cao J, Huang X, Chen Z, Xu P, Sun G, Xu J, Lv J (2019). Circular RNA circNRIP1 acts as a microRNA-149-5p sponge to promote gastric cancer progression via the AKT1/mTOR pathway. Mol Cancer.

[22] Wang S, Tang D, Wang W, Yang Y, Wu X, Wang L, Wang D (2019). circLMTK2 acts as a sponge of miR-150-5p and promotes proliferation and metastasis in gastric cancer. Mol Cancer.

[23] Zhang J, Liu H, Hou L, Wang G, Zhang R, Huang Y, Chen X, Zhu J (2017). Circular RNA_LARP4 inhibits cell proliferation and invasion of gastric cancer by sponging miR-424-5p and regulating LATS1 expression. Mol Cancer.

[24] Liu H, Liu Y, Bian Z, Zhang J, Zhang R, Chen X, Huang Y, Wang Y, Zhu J (2018). Circular RNA YAP1 inhibits the proliferation and invasion of gastric cancer cells by regulating the miR-367-5p/p27 Kip1 axis. Mol Cancer.

[25] Zhang J, Hou L, Liang R, Chen X, Zhang R, Chen W, Zhu J (2019). CircDLST promotes the tumorigenesis and metastasis of gastric cancer by sponging miR-502-5p and activating the NRAS/MEK1/ERK1/2 signaling. Mol Cancer.

[26] Yang X, Zhang S, He C, Xue P, Zhang L, He Z, Zang L, Feng B, Sun J, Zheng M (2020). METTL14 suppresses proliferation and metastasis of colorectal cancer by down-regulating oncogenic long non-coding RNA XIST. Mol Cancer.

[27] Xu J, Wan Z, Tang M, Lin Z, Jiang S, Ji L, Gorshkov K, Mao Q, Xia S, Cen D (2020). N6-methyladenosine-modified CircRNA-SORE sustains sorafenib resistance in hepatocellular carcinoma by regulating β-catenin signaling. Mol Cancer.

[28] Liu X, Xiao M, Zhang L, Li L, Zhu G, Shen E, Lv M, Lu X, Sun Z (2021). The m6A methyltransferase METTL14 inhibits the proliferation, migration, and invasion of gastric cancer by regulating the PI3K/AKT/mTOR signaling pathway. J Clin Lab Anal.

[29] Dong L, Chen C, Zhang Y, Guo P, Wang Z, Li J, Liu Y, Liu J, Chang R, Li Y (2021). The loss of RNA N6-adenosine methyltransferase Mettl14 in tumor-associated macrophages promotes CD8+ T cell dysfunction and tumor growth. Cancer Cell.

[30] Du Y, Hou G, Zhang H, Dou J, He J, Guo Y, Li L, Chen R, Wang Y, Deng R (2018). SUMOylation of the m6A-RNA methyltransferase METTL3 modulates its function. Nucleic Acids Res.

[31] Li X, Tian G, Wu J (2021). Novel circGFRα1 Promotes Self-Renewal of Female Germline Stem Cells Mediated by m6A Writer METTL14. Front Cell Dev Biol..

[32] Tanaka T, Okada R, Hozaka Y, Wada M, Moriya S, Satake S, Idichi T, Kurahara H, Ohtsuka T, Seki N (2020). Molecular pathogenesis of pancreatic ductal adenocarcinoma: impact of miR-30c-5p and miR-30c-2-3p regulation on oncogenic genes. Cancers (Basel).

[33] Shukla K, Sharma AK, Ward A, Will R, Hielscher T, Balwierz A, Breunig C, Münstermann E, König R, Keklikoglou I (2015). MicroRNA-30c-2-3p negatively regulates NF-κB signaling and cell cycle progression through downregulation of TRADD and CCNE1 in breast cancer. Mol Oncol.

[34] Mathew LK, Lee SS, Skuli N, Rao S, Keith B, Nathanson KL, Lal P, Simon MC (2014). Restricted expression of miR-30c-2-3p and miR-30a-3p in clear cell renal cell carcinomas enhances HIF2α activity. Cancer Discov.

[35] Xu Q, Xu Z, Zhu K, Lin J, Ye B (2021). LINC00346 Sponges miR-30c-2–3p to Promote the Development of Lung Adenocarcinoma by Targeting MYBL2 and Regulating CELL CYCLE Signaling Pathway. Front Oncol.

[36] Zhang HD, Jiang LH, Hou JC, Zhou SY, Zhong SL, Zhu LP, Wang DD, Yang SJ, He YJ, Mao CF (2018). Circular RNA hsa_circ_0072995 promotes breast cancer cell migration and invasion through sponge for miR-30c-2-3p. Epigenomics.

[37] Tang CT, Liang Q, Yang L, Lin XL, Wu S, Chen Y, Zhang XT, Gao YJ, Ge ZZ (2018). RAB31 Targeted by MiR-30c-2-3p Regulates the GLI1 Signaling Pathway, Affecting Gastric Cancer Cell Proliferation and Apoptosis. Front Oncol.

[38] Qi Z, Zhang T, Song L, Fu H, Luo H, Wu J, Zhao S, Zhang T, Guo L, Jin L (2020). PRAS40 hyperexpression promotes hepatocarcinogenesis. EBioMedicine.

[39] Xuefang Z, Ruinian Z, Liji J, Chun Z, Qiaolan Z, Jun J, Yuming C, Junrong H (2020). miR-331-3p Inhibits Proliferation and Promotes Apoptosis of Nasopharyngeal Carcinoma Cells by Targeting elf4B-PI3K-AKT Pathway. Technol Cancer Res Treat.

