# Supplementary material for: METTL14-mediated m6A modification of circORC5 suppresses gastric cancer progression by regulating miR-30c-2-3p/AKT1S1 axis
Source: Mol Cancer. 2022 Feb 14;21:51. doi: 10.1186/s12943-022-01521-z (PMC8842906; doi:10.1186/s12943-022-01521-z)
Supplement: Supplementary file 1 — Additional file 1: Table S1. The sequences of the primers. Table S2. The correlation of METTL14 expression with clinicopathologic characteristics of GC patients. Table S3. Cox regression analysis of METTL14 expression as survival predictor. [file 12943_2022_1521_MOESM1_ESM.docx]

**Supplementary Figure legends**

**Supplementary Figure S1:** RT-qPCR analysis of the transfection efficiency of si-circORC5 in MGC-803 and AGS cells.

**Supplementary Figure S2:** The transfection efficiency of miR-30c-2-3p mimics and its effects on circORC5 expression were measured by RT-qPCR in MGC-803 and AGS cells.

**Supplementary Figure S3:** Schematic representation of potential binding sites between miR-30c-2-3p and AKT1S1/ EIF4B.

**Supplementary Tables**

**Table S1.** The sequences of the primers

| Gene | Primer | Sequence (5'-3') |
| --- | --- | --- |
| U6 | Forward | CGCTTCGGCAGCACATATAC |
|  | Reverse | AAATATGGAACGCTTCACGA |
| homo GAPDH | Forward | TCAAGAAGGTGGTGAAGCAGG |
|  | Reverse | TCAAAGGTGGAGGAGTGGGT |
| Homo m^6^A | Forward | CTGAGCTGAACATGACCTTCC |
|  | Reverse | TGGAGCGAGTAGAGTGGATGT |
| hsa_circORC5 | Forward | GTTGGCCATGACGATCAGC |
|  | Reverse | ACTCTGCTGTCCACGATACT |
| hsa_circ_0047481 | Forward | ACTCCTTGGAGCTGTTATCCTC |
|  | Reverse | TCACCATGTGTCCTTGTCGA |
| hsa_circ_0030632 | Forward | CATTTCACAGAACTTCCCCATAA |
|  | Reverse | TTTTCCGTGAGCTTGGTTTC |
| homo METTL14 | Forward | GAGATTGCAGCACCTCGATC |
|  | Reverse | TGCTACGCTTCACAGTTCCT |
| hsa-miR-30c-2-3p | Forward | TGCGCCTGGGAGAAGGCTGTTT |
|  | Reverse | CCAGTGCAGGGTCCGAGGTATT |
| ORC5 | Forward | TGCTTTGAGCCGTTTGTCTTA |
|  | Reverse | TGTAGGCAGCATAGAAATCAGC |
| EIF4B | Forward | AAGGCCAAACTGGGAACTCTA |
|  | Reverse | CCCTCATTTTCATCTTCACCA |
| AKT1S1 | Forward | CACACAGCAGTACGCCAAGT |
|  | Reverse | GGAAGTCGCTGGTGTTAAGC |

**Table S2** The correlation of METTL14 expression with clinicopathologic

characteristics of GC patients

| Variables | Cases  (n) | METTL14 | | *P* value |
| --- | --- | --- | --- | --- |
|  |  | High | Low |  |
| Total | 292 | 189 | 103 |  |
| *Age (years)* |  |  |  |  |
| ≥60 | 197 | 122 | 75 |  |
| <60 | 95 | 67 | 28 | 0.191 |
| *Gender* |  |  |  |  |
| Male | 187 | 119 | 68 |  |
| Female | 105 | 70 | 35 | 0.702 |
| *Pathological stage* |  |  |  |  |
| Ⅰ/Ⅱ | 139 | 93 | 46 |  |
| Ⅲ/Ⅳ | 153 | 96 | 57 | 0.465 |
| *T stage* |  |  |  |  |
| T1/T2 | 74 | 50 | 24 |  |
| T3/T4 | 218 | 139 | 79 | 0.577 |
| *N stage* |  |  |  |  |
| Negative | 99 | 69 | 30 |  |
| Positive | 193 | 120 | 73 | 0.244 |
| *M stage* |  |  |  |  |
| Negative | 263 | 171 | 92 |  |
| Positive | 29 | 18 | 11 | 0.838 |

**Table S3** Cox regression analysis of METTL14 expression as survival predictor

| Variables | Univariate Cox regression analysis | |  | Multivariate Cox regression analysis | |
| --- | --- | --- | --- | --- | --- |
|  | RR (95% CI) | *P* value |  | RR (95% CI) | *P* value |
| *Age (years)* |  |  |  |  |  |
| <60 vs. ≥60 | 1.757 (1.111 to 2.779) | 0.016 |  | 1.801 (1.134 to 2.860) | 0.013 |
| *Gender* |  |  |  |  |  |
| Male vs. Female | 1.257 (0.820 to 1.928) | 0.293 |  | NA | NA |
| *Pathological stage* |  |  |  |  |  |
| Ⅲ/Ⅳ vs.Ⅰ/Ⅱ | 1.852 (1.212 to 2.381) | 0.004 |  | 1.690 (1.061 to 2.690) | 0.027 |
| *T stage* |  |  |  |  |  |
| T3+T4 vs. T1+T2 | 1.755 (1.037 to 2.973) | 0.036 |  | 1.427 (0.802 to 2.540) | 0.227 |
| *N staging* |  |  |  |  |  |
| Positive vs. Negative | 1.457 (0.929 to 2.284) | 0.101 |  | NA | NA |
| *M stage* |  |  |  |  |  |
| Positive vs. Negative | 1.642 (0.915 to 2.949) | 0.097 |  | NA | NA |
| *METTL14 expression* |  |  |  |  |  |
| High VS. Low | 0.526 (0.353 to 0.785) | 0.002 |  | 0.562 (0.375 to 0.840) | 0.005 |

NA: not analyzed
