# Supplementary figures and images for: METTL14-mediated m6A modification of circORC5 suppresses gastric cancer progression by regulating miR-30c-2-3p/AKT1S1 axis
Source: Mol Cancer. 2022 Feb 14;21:51. doi: 10.1186/s12943-022-01521-z (PMC8842906; doi:10.1186/s12943-022-01521-z)

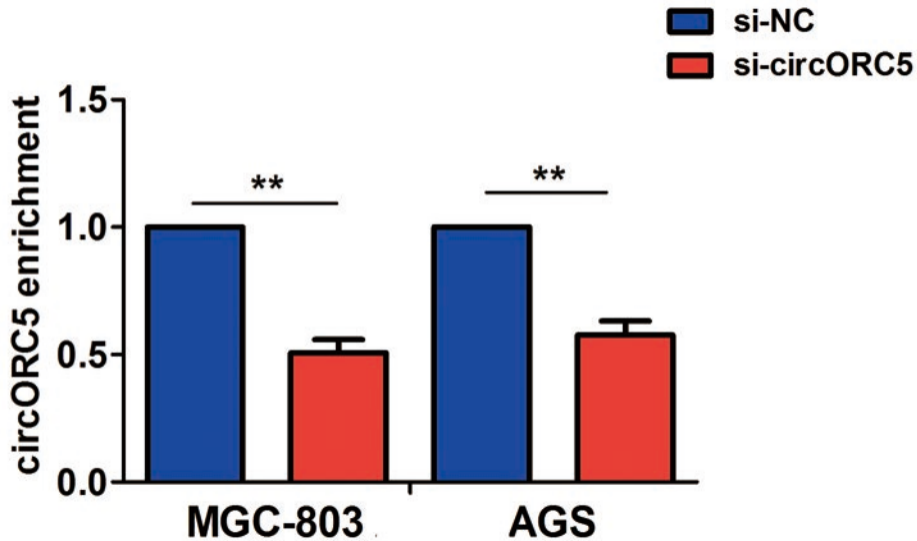

Supplement: Supplementary file 2 — Additional file 2: Supplementary Figure S1. RT-qPCR analysis of the transfection efficiency of si-circORC5 in MGC-803 and AGS cells. [file 12943_2022_1521_MOESM2_ESM.pdf]

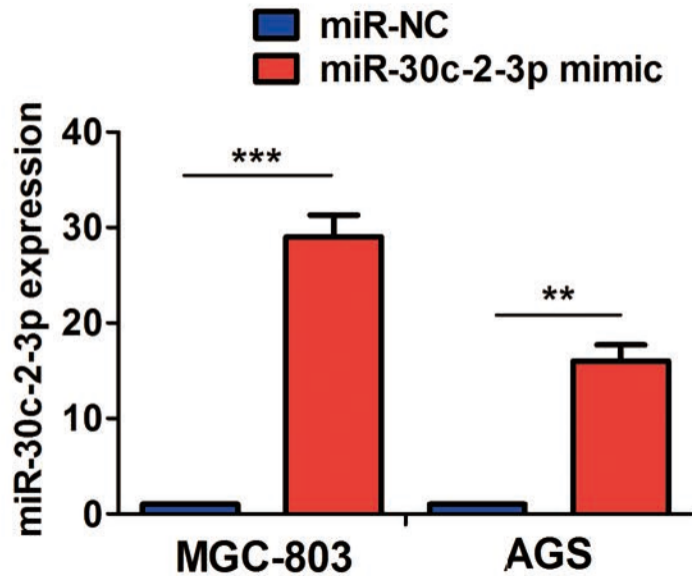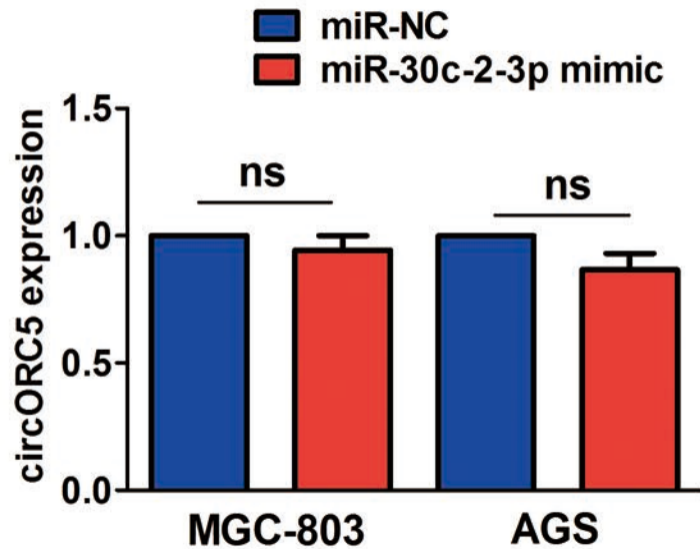

Supplement: Supplementary file 3 — Additional file 3: Supplementary Figure S2. The transfection efficiency of miR-30c-2-3p mimics and its effects on circORC5 expression were measured by RT-qPCR in MGC-803 and AGS cells. [file 12943_2022_1521_MOESM3_ESM.pdf]

**A**

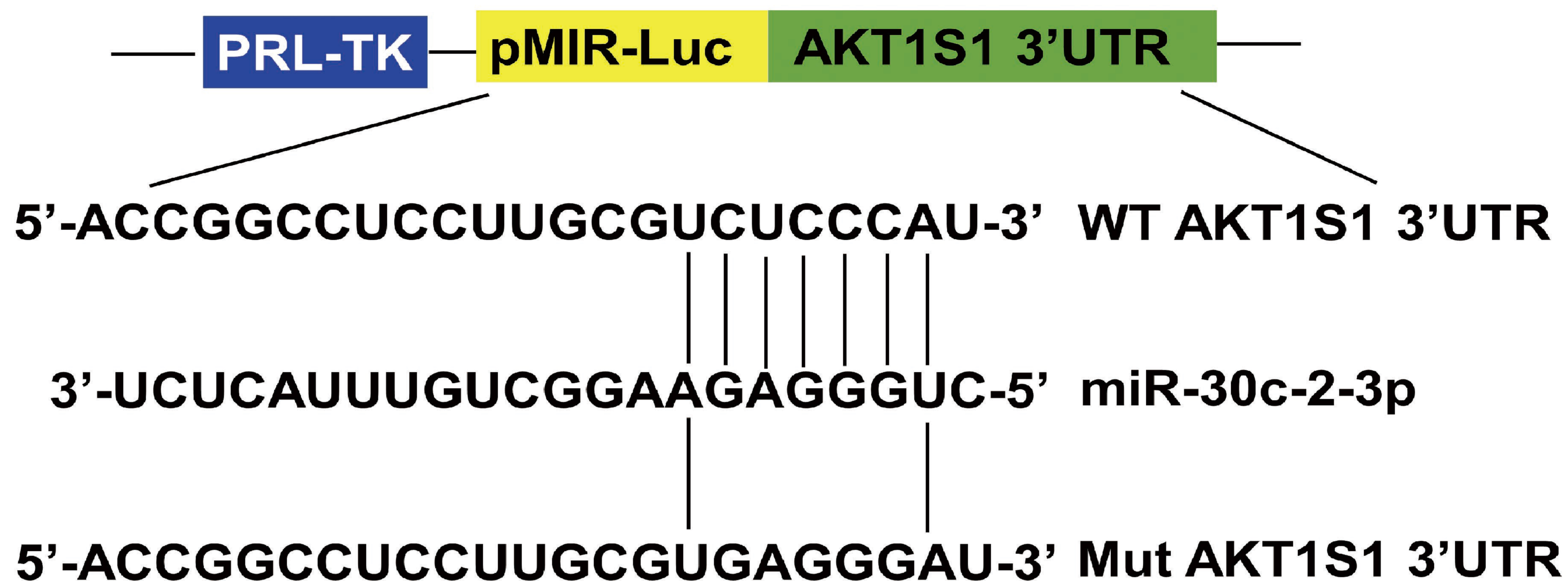

**B**

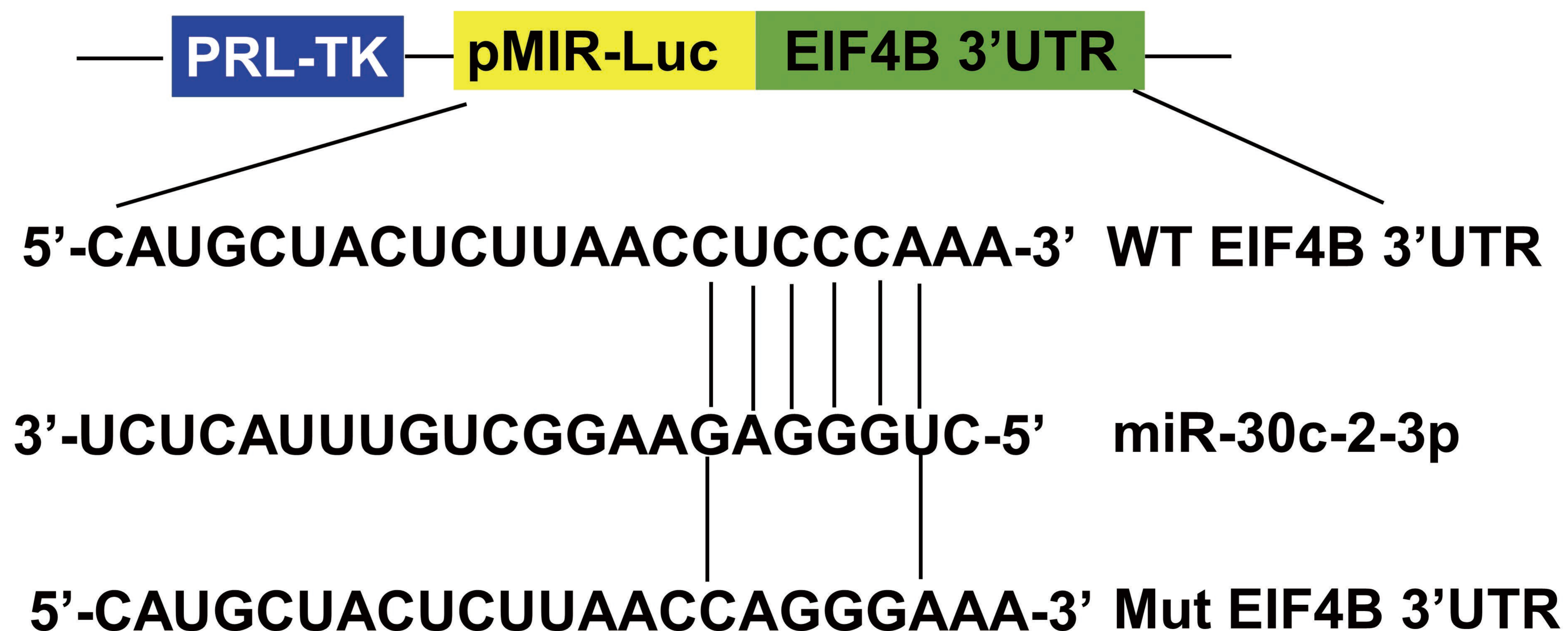

Supplement: Supplementary file 4 — Additional file 4: Supplementary Figure S3. Schematic representation of potential binding sites between miR-30c-2-3p and AKT1S1/ EIF4B. [file 12943_2022_1521_MOESM4_ESM.pdf]
